# No small feat: microRNA responses during vocal communication in songbirds

**DOI:** 10.1186/1741-7007-9-35

**Published:** 2011-05-31

**Authors:** Claudio V Mello, Peter V Lovell

**Affiliations:** 1 Department of Behavioral Neuroscience, Oregon Health and Science University, 3181 Sam Jackson Park Rd L470, Portland, OR 97239, USA

## Abstract

Simply hearing the song produced by another bird of the same species triggers the regulation of microRNAs (miRNAs) in high-order auditory parts of the zebra finch brain. Some of the identified miRNAs appear to be unique to birds, possibly to songbirds. These findings, reported in *BMC Genomics*, highlight the complexities of gene regulation associated with vocal communication and point to possible key regulators of song-triggered gene networks.

**See research article:**http://www.biomedcentral.com/1471-2164/12/277

## Brain gene regulation in vocal communication

**Figure 1:**
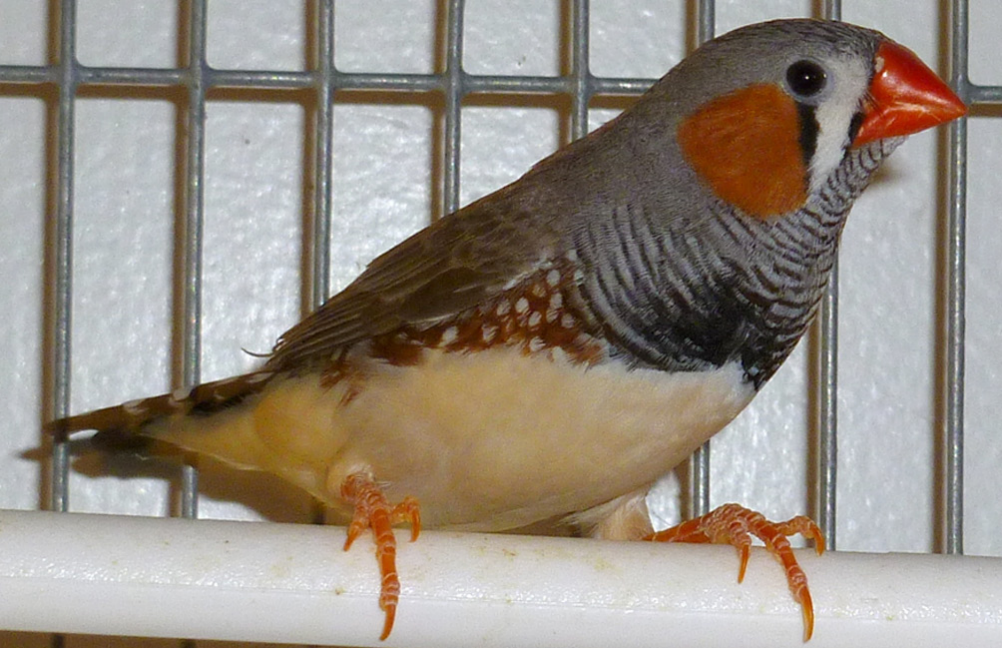
**Zebra finch**. Zebra finches (*Taeniopygia guttata*), like this adult male, have contributed much to our understanding of the anatomical, physiological, neurochemical, and molecular properties of neural circuits that are involved in the perception, production and learning of birdsong.

Immersed in a vast ocean of sounds our brains seek to process and memorize only those sounds that are most relevant to us. Thus, we tend to pay a lot of attention to vocal signals produced by other members of our own species, particularly those that convey information critical to social interaction, reproduction and survival. We are talking, of course, about the sounds that comprise speech and language. Remarkably, only a few animals communicate through a system of vocalizations with characteristics that resemble the complexities and capabilities of human speech. Songbirds (more technically, Passeriformes oscines) represent one such group. They not only communicate through complex vocal signals (songs), but also like humans they learn them through vocal imitation [1]. In many respects this capability mirrors the acquisition of speech in humans, and has made songbirds an exquisite model organism for unraveling the neuronal basis of vocal and speech learning ([2] and references therein). Among the numerous basic insights contributed by songbird research is the demonstration that complex gene networks are rapidly regulated in the brain when songbirds hear song or engage in singing [2]. Microarray studies, in particular, have revealed that song exposure influences the expression of hundreds of genes in the auditory forebrain of the zebra finch, *Taeniopygia guttata *([3] and Lovell and Mello, unpublished observations) (Figure 1). Chief among these, the activity-regulated immediate early genes (IEGs) *zenk *(also known as *zif-268*, *egr-1 *or *ngfi-a*), *fos*, *jun *and *arc *suggest a dynamic link between the perceptual processing and memorization of birdsong, and underlying transcriptional networks that regulate properties of brain circuits [4,5]. Now, a study by Gunaratne *et al. *[6] extends these findings, revealing that microRNAs (miRNAs), a class of small non-coding RNAs that may serve as control points in transcriptional networks [7,8], are also a major component of the genomic response to song. This study may thus begin to elucidate some of the key regulatory mechanisms that coordinate the genomic response to learned vocal communication signals.

## What can we learn from a squeaky little bird?

The zebra finch has long been a favorite among songbird neurobiologists despite having a song that is rather shrill and repetitive to the human ear - a far cry from the pleasant melodies that recount the beauty and musicality of birdsong. However unharmonious, the structure and development of zebra finch song are well characterized, making the process of vocal imitation relatively easy to quantify. Moreover, the brain circuits involved in song processing, learning and production are known in exquisite detail, greatly facilitating the search for physiological properties and mechanisms underlying song acquisition [2]. More recently, the completed sequencing, assembly and annotation of the genome [9], as well as resources such as comprehensive brain cDNA/EST collections, microarrays, and transgenic efforts (developed by songbird research labs at UIUC, Rockefeller University, Duke University, MIT, and OHSU, among others), point to the zebra finch as the species of choice for uncovering molecular genetic mechanisms underlying song learning. It should be no surprise, therefore, that studies of brain gene regulation in a behaviorally relevant context have become quite refined in this species.

## Birdsong: a confluence between natural behavior and genomic regulatory mechanisms

The finding that song regulates brain gene expression provides one of the earliest and clearest examples of gene expression in a vertebrate organism driven in response to natural stimuli with behavioral relevance. Analysis of the *zenk *gene in particular has been instrumental in the mapping of brain circuits involved in the perceptual processing, learning and production of birdsong [4], and has yielded much information about the internal organization of these brain areas. Within this context, the caudomedial portion of the songbird brain (referred to by Gunaratne *et al. *as the auditory lobule (AL)), which is arguably analogous to parts of the mammalian auditory cortex, has risen to prominence for its putative role in the perceptual processing and memorization of birdsong [10]. Recently, synapsins have also been identified as late song-regulated genes and potential regulatory targets of the transcription factor zenk [11]. Interestingly, synapsin genes and *arc *, among the main song-regulated genes identified so far, act at the pre-synaptic and post-synaptic levels, respectively, suggesting that song-regulated gene networks may largely exert actions at the level of synapses, where they could be regulating levels of experience-dependent plasticity. Indeed, zenk, arc and synapsins have all been implicated in different aspects of synaptic physiology or plasticity in mammals [12,13].

Despite these advances, our basic understanding of the molecular components and key regulatory features of song-regulated gene programs are still relatively limited. To address this issue, songbird investigators have begun to turn to more high-throughput methods, such as microarray analysis and next-generation RNA sequencing, to identify specific genes regulated in the AL during hearing, or within nuclei of the song system during singing. Hundreds of genes have been implicated, highlighting the staggering complexity of song-regulated cascades [4,9]. Amid this apparent chaos, however, some order is required, and it has become mission critical to find mechanisms that may coordinate the gene networks underlying the processing and production of birdsong. In step the miRNAs.

## Small RNAs that pack a big punch

Small non-coding miRNAs have rapidly emerged as an important feature of gene regulatory cascades in different tissues, including the brain [8], and thus seemed obvious, if unexplored, targets for investigation in songbirds. Gunaratne *et al. *hypothesized that miRNAs might be involved in the songbird brain's response to hearing song, which could have important consequences for song processing and memorization. To investigate this possibility, they first identified the complement of miRNAs present in the zebra finch genome. Here some interesting surprises emerged. Beyond finding more than a hundred conserved miRNAs, they also discovered 48 novel RNAs that met stringent criteria for being classified as miRNAs. Further, many of these may be unique to songbirds as they are not found in the chicken genome, suggesting that the evolutionary processes that led to the emergence of the songbird lineage may have been associated with the rise of unique miRNAs, an exciting concept that will eventually be tested as other avian genomes become available.

More directly related to their original goal, the authors next made use of song playbacks to demonstrate that several miRNAs are either up- or down-regulated in the AL when birds are listening to conspecific song. Thus, miRNA expression is actively regulated by hearing birdsong, providing a clear demonstration that this class of genes is also sensitive to relevant behavioral experience. It is particularly intriguing that one of the most prominently regulated miRNAs resides on the Z chromosome, present in two copies in males, the homogametic sex in birds. Although the relevance of this observation is not entirely clear, it is interesting to note the song system and singing behavior are highly dimorphic in zebra finches, occurring in males but not females (reviewed in [2]). This suggests the intriguing possibility that a miRNA might be linked to the regulation of some of the dimorphic features of the vertebrae brain. The authors have also identified candidate targets of the newly discovered miRNAs; some of these appear to be involved in processes like cell proliferation and neurite outgrowth, suggesting they may play key roles in establishing and/or maintaining brain circuitry for the production and processing of birdsong. Further elucidation of the functional role of miRNAs in vocal learning should be a highly interesting and rewarding avenue for future investigation.

## Realizing the potential of the songbird genome

The rush of recent publications documenting the complexities of the zebra finch genome suggest that the songbird community is well on its way to realizing the potential of using genomics tools for understanding how the environment, experience, and the genome interact to produce the complex and adaptive behavior of vocal learning in birds. In the specific study discussed here, novel data have been brought to light about the participation of miRNAs in the brain's response to vocal communication signals, highlighting the fact that the brain transcriptome is highly dynamic, even more so than originally suspected. In practical terms, these studies also illustrate well how the willingness of the NIH to fund and promote research in non-traditional organisms is paying off, especially in studies that capture aspects of human behavior such as speech and language that are not represented in more traditional genetic organisms. As songbirds sing their way through life, we can only hope that this trend will continue and that more fundamental insights will come to light on the interface between birdsong, the genome and the brain.
